# A novel Monte Carlo simulation procedure for modelling COVID-19 spread over time

**DOI:** 10.1038/s41598-020-70091-1

**Published:** 2020-08-04

**Authors:** Gang Xie

**Affiliations:** Research Office, Charles Sturt University, Wagga Wagga, NSW Australia

**Keywords:** Diseases, Health care, Medical research, Risk factors

## Abstract

The coronavirus disease 2019 (COVID-19) has now spread throughout most countries in the world causing heavy life losses and damaging social-economic impacts. Following a stochastic point process modelling approach, a Monte Carlo simulation model was developed to represent the COVID-19 spread dynamics. First, we examined various expected performances (theoretical properties) of the simulation model assuming a number of arbitrarily defined scenarios. Simulation studies were then performed on the real COVID-19 data reported (over the period of 1 March to 1 May) for Australia and United Kingdom (UK). Given the initial number of COVID-19 infection active cases were around 10 for both countries, the model estimated that the number of active cases would peak around 29 March in Australia (≈ 1,700 cases) and around 22 April in UK (≈ 22,860 cases); ultimately the total confirmed cases could sum to 6,790 for Australia in about 75 days and 206,480 for UK in about 105 days. The results of the estimated COVID-19 reproduction numbers were consistent with what was reported in the literature. This simulation model was considered an effective and adaptable decision making/what-if analysis tool in battling COVID-19 in the immediate need, and for modelling any other infectious diseases in the future.

## Introduction

The coronavirus disease 2019 (COVID-19) epidemic began in Wuhan city, China in December, 2019^1^. WHO officially declared COVID-19 a pandemic in early March 2020 and COVID-19 has now spread throughout most countries in the world^2^ causing heavy life losses and damaging social-economic impacts^3^. The COVID-19 outbreak has actually turned a regional public health threat into a global social-economic crisis.

This study aimed at developing a COVID-19 spread dynamics based Monte Carlo simulation model that could be used as a decision making tool in battling COVID-19 in the immediate need, and for modelling any other infectious diseases in the future. Same as those compartmental disease models within the Susceptible-Infectious-Recovered (SIR) framework^3–5^, the proposed model captured population changes in each cohort. However, the approach for modelling the dynamic processes of virus/disease transmission is different. In this study, the progression of COVID-19 was characterised by a Monte Carlo simulation model which is similar to a stochastic point process model^6,7^. By treating each individual in a population as a random point, the rationale of the new model was much intuitive and easy to interpret. The modelling processes were implemented using the popular statistical software R^8^. Using this article as a user guide, the application of the new model would be straight forward for an experienced R user. The outputs of the new model included the estimated daily number of newly confirmed cases and the daily number of the infection active cases (i.e., new cases and the carry-over infectious cases) over a specified observation period. The model outputs therefore contained the information for answering the most essential decision making questions such as when the number of the active COVID-19 cases would peak, how long the number of the active cases would fall back to a specified level, what would be the total number of confirmed cases by the end of the COVID-19 outbreak, etc. The most important parameter of the simulation model was the average/expected number of cases that a single infectious person would infect over the course of this person’s infectious period (i.e., the reproduction number as usually reported in literature, however, primarily referred to as the infection rate parameter ‘Rt’ in this article). It was a matter of fact that the determination of the infection rate Rt over an observation period depended on many factors that at least including goodness-of-fit of the observed number of confirmed COVID-19 cases, interpretation of the impact of government interventions/policies, the capacity of health care system, and evaluation of the potential consequences due to public responses to government policies and induced people’s behaviour changes^1,3,9^. By statistically capturing the most essential COVID-19 spread mechanism, this simulation model should at least provide a valid and effective alternative to those SIR framework based models for what-if analysis in decision making to minimise the losses caused by COVID-19 outbreak.

## Method and technical details of the simulation model

The COVID-19 spread dynamics was characterised by a few key parameters in building the simulation model. The key parameters and assumptions were: (1) the infection rate parameter ‘Rt’; (2) the actual number of people being infected by each of the infection active cases is determined by a Poisson distribution with mean Rt; the exact number of days for someone getting infected follows a negative binomial distribution with the mean/expected time (parameter ‘muT’) in days. (3) The limit of the number of people and the immunity proportion in a study population could be pre-determined by the user. (4) Other initial conditions including the initial number of infectious persons, the observation period (in days), and the simulation period (in days) of a simulation study.

**Figure 1:**
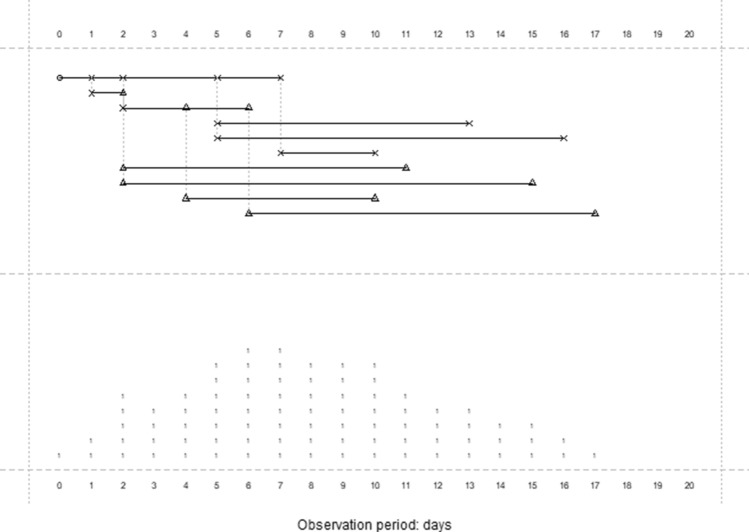
A schematic of a stochastic point process model with a hypothetic population of 10 people (10 horizontal lines in the top panel): the virus transmission initiated from one person (represented by the initial round dot point) who then infected five people (represented by the cross points); two of the five secondary infectious individuals were able to infect the third generation of infected people (represented by the triangle points). The bottom panel displayed the number of the active cases pattern.

A stochastic point process conceptual model was best illustrated with a graph as shown in Fig. 1, assuming a hypothetic micro-population of 10 people. The observation period was 20 days. The COVID-19 spread processes started from one person (represented by the initial round dot point) who infected five people (represented by the cross points). The number of people being infected was modelled by a Poisson distribution with parameter Rt which could be different in different stage of the observation period. We assume that a minimum of one day is needed for a newly infected person to start transmitting the COVID-19 to the next generation of infected people. The infection times are independent of each other so that more than one person could be infected from the same infection active person on the same day. The exact number of days for someone to be get infected followed a negative binomial distribution with the mean/expected time (parameter ‘muT’) in days. Once the Poisson distribution generated number of infected people was reached, the source infection active person would be excluded from the transmission process (e.g., assuming the source infectious person is either cured or died, or at least in a condition of incapable of causing infection of other people, therefore no longer staying in the infection active case cohort). If an infected person had no one else to transmit the virus, this person would be excluded from the active case cohort after muT days on average. In Fig. 1, the top panel plot showed us how many and when of the infection cases would happen for a micro-population of 10 people within an observation period of 20 days; the bottom panel displayed the corresponding pattern of the number of the active cases (i.e., the daily number of people who were actively infectious).

The simulation model was implemented in R^8^ and the r code was provided in the Supplementary informmation for readers’ information (the full set of the r code for reproduction of all the analysis results presented in this article would be available upon request). The simulation model had the following format for usage in R environment^8^.

TransSimu (days = 300, nd = 30, Rt = rr, muT = 4, sizeV = 1, limit = 1,000,000, pp = 0.001, n0 = 1).

There were eight parameters/argument terms in this R function and definitions of these argument terms were given in Table 1.

**Table 1:** Definitions of the argument terms for the simulation function TransSimu().

Input parameter	Definition of the input parameter
days	Observation period (in days) of a simulation study
nd	Simulation period (in days) of a simulation study
Rt	Average reproduction/infection rate (i.e., the expected number of secondary cases that each existing infectious case will generate); Rt is a function of time/day
muT	Average/expected number of days for an existing infectious person to infect a susceptible person in the population; mean parameter for the negative binomial distribution^10,11^
sizeV	The dispersion parameter for the negative binomial distribution so that variance = muT + (muT)^2^/sizeV^10,11^
limit	The study/target population size
pp	The proportion of people with immunity in the population
n0	The initial number of infectious persons prior to the observation/simulation period

Due to the uncertainty introduced by probability distributions employed for determining the number of infection cases and the time at which such event would occur, we should allow the observation period (‘days’) relatively longer than the simulation period (‘nd’). For modelling COVID-19 data, it was found that nd = 100 was enough to obtain a stabilized simulation results. The length of Rt should be as least as large as the nd value. This would be better explained through an example.

Suppose now we had a hypothetic infection rate pattern as rr = c(rep(2.8,30), 2,2,1.5,1.5, 1.2,1.2,rep(0.8,4),rep(0.5,10)) , hence length of Rt is 50. If we tried to run the simulation model as TransSimu(nd = 100,Rt = rr) in R, an error message would be returned in the R console window: Error in TransSimu(nd = 100, Rt = rr): the length of Rt should not be smaller than nd.

Since Monte Carlo simulation processes involve the random number generation which means the simulation analysis results would be subject to random variation due to different starting points defined intrinsically by a selected random seed value^10,12^. Therefore, when we would compare different scenarios of the disease transmission using the simulation model, the same random seed needed to be specified for each scenario for a valid comparison.

The determination of the infection rate patterns was a mixed exercise procedure involving using reported reproduction numbers (referred to as infection rates in this article) as a base line to start, performing statistical optimization (e.g., least square curve fit of the observed data), and incorporating personal judgement and interpretation of the government intervention effects. For example, curve fitting could only be done for those days the data of confirmed COVID-19 cases were available which were 62 days out of the 100-day model simulation period in this study. Therefore, the remaining 38 of the total 100 Rt = rr values were primarily the results of subjective judgement and assumptions. It was in this sense we considered that primarily what this Monte Carlo simulation model could do for us was of the nature of what-if analysis for decision making.

To count for the uncertainty in a Monte Carlo simulation study, a bootstrap approach^10,13^ was followed to obtain the estimated median and interquartile values of the number of the infection active cases and the confirmed daily new cases over the observation period (hence the total number of confirmed cases could easily be derived). Each simulation model was run for 1,000 times and the median values were considered as the most likely estimation and the uncertainty level was characterised by the interquartile range (i.e., the range between the 25th percentile and 75th percentile of the 1,000 values).

In order to show how to use this simulation model to count for the impact of population size and immunity level, three hypothetical infection rate patterns were assumed for simulation study. The comparison of these three infection rate patterns was to show the simulation model performance in the case when the population size limit would be reached and the cohort with immunity should be counted for in the disease transmission processes. This study also showed under what condition the number of the infection active case curve could be flattened.

The outputs of the simulation model included three pieces of information: the estimated daily number of the active cases; the estimated daily number of the new cases; and a single total number of all confirmed cases. Therefore, summing up all the estimated number of the daily new confirmed cases should equal exactly the overall total number of the confirmed cases.

## Results

### Simulation results and comparison of 12 hypothetical disease transmission scenarios

In this study, we first examined the model theoretical performances using a few reasonable but hypothetic infection rate patterns. Three such patterns expressed in r code format were given below.

rr = c(rep(3,30), 2,2,1.5,1.5, 1.2,1.2,rep(0.8,4),rep(0.5,10),rep(0.1,50)).

rr = c(rep(3,25), 2,2,1.5,1.5, 1.2,1.2,rep(0.8,4),rep(0.5,10),rep(0.1,55)).

rr = c(rep(2.5,30), 2,2,1.5,1.5, 1.2,1.2,rep(0.8,4),rep(0.5,10),rep(0.1,50)).

Literature reported that the typical range for Rt values was between 2 to 4 for COVID-19 spread without intervention^1,3,9,14^. Assuming the simulation period started before any government interventions, therefore, Rt values were set to 3 for the first 30 days in the pattern 1 and then decreasing gradually over the subsequent stages of the 100 days presumably due to the effect of government interventions. In pattern 2, we would like to check how the outbreak outcomes would change if the government’s intervention enforced 5 days earlier; further in pattern 3, we examined how different it could be if the starting Rt values were lower than 3 (e.g., 2.5) given other things equal. As indicated in Table 1, there were two parameters (muT and sizeV) for defining a negative binomial distribution^10,11^ which was used for describing the time interval for a susceptible person getting infected. In the study, we also compared different scenarios by examining different muT and sizeV values. The three different simulation settings that examined were: TransSimu(nd = 100, muT = 4, sizeV = 1); TransSimu(nd = 100, muT = 4, sizeV = 0.9); and TransSimu(nd = 100, muT = 3.6, sizeV = 1).

**Figure 2:**
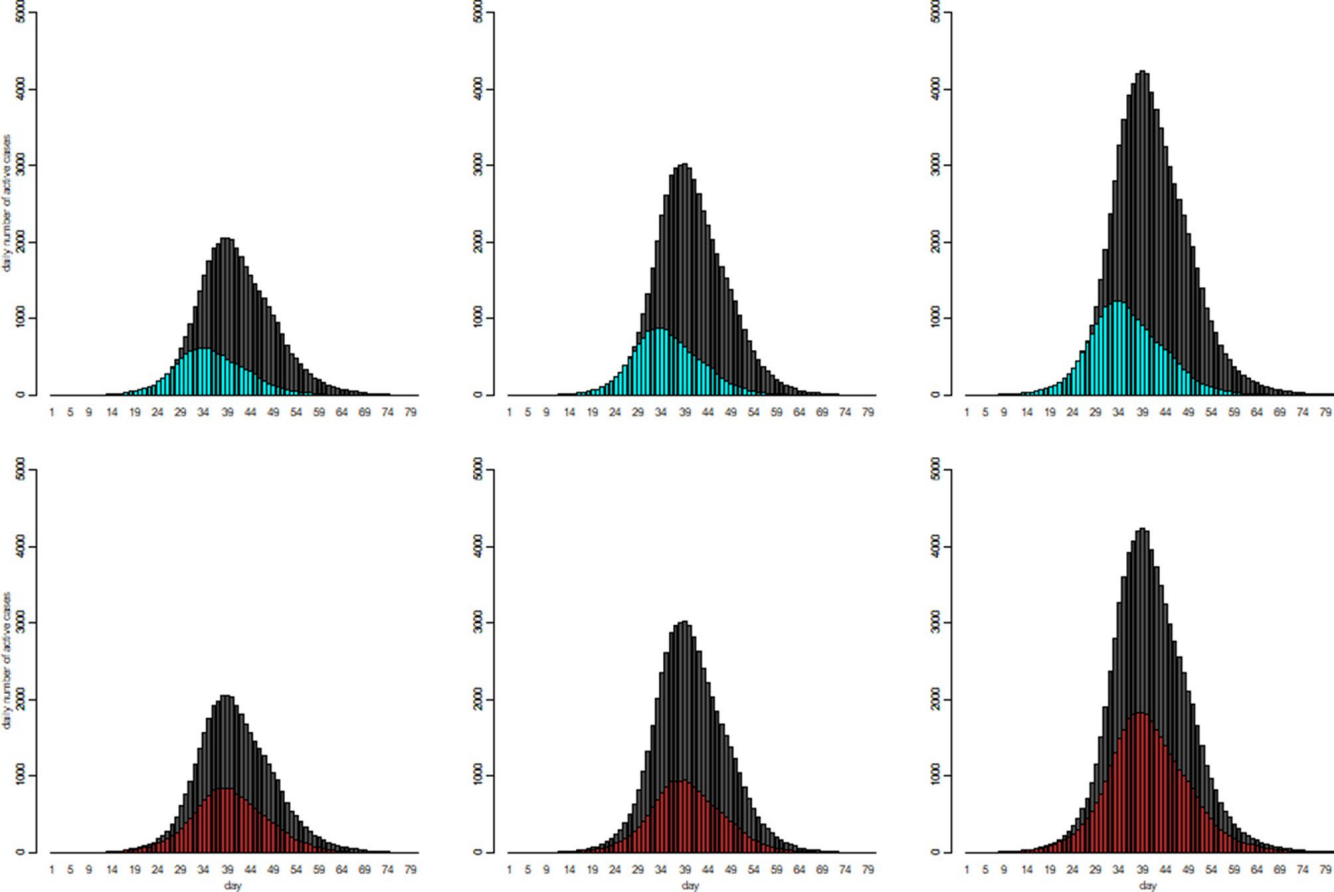
The patterns of the number of the active cases over the observation period with respect to 12 disease transmission scenarios which consists of the combinations of three infection rate patterns and two muT levels and two sizeV levels.

Therefore, we finally ended up with a total of 12 hypothetical disease transmission scenarios for us to investigate/compare using the proposed simulation model and the results were presented in Fig. 2.

The interpretations of the graphical outcomes of Fig. 2 were as follows. The three panels in the top row showed that by only five days earlier of enforcing intervention, the number of the active cases would peak earlier accordingly with a much lower peak value (black curves versus blue curves). The three panels in the bottom row showed that by reducing Rt from 3 to 2.5 for the first 30 days, the number of the active cases would reach the peak on the same day but with a much lower peak value (black curves versus red curves). The comparison of the panels in the second column versus the first column showed that reducing the muT values (3.6 versus 4 days) would increase the peak level quite substantially. The comparison of the panels in the third column versus the first column showed that reducing the sizeV values (0.9 versus 1, a smaller sizeV value implies a larger variance) would increase the peak level even further. By comparing panels in columns three and two seemed indicating the impact on the peak level due to the change (10%) in muT was somehow smaller than that due to change (10%) in sizeV. Therefore, in summary, this part of the simulation study showed that, as expected/implied by the underlying theory, a higher Rt or a smaller muT would result in a higher number of infected people keeping other things equal.

### Simulation study results on Australia and UK data

The simulation model was then tested with two real COVID-19 data sets: the confirmed COVID-19 cases reported for Australia and United Kingdom (UK) over the period 1 March to 1 May 2020^2^. The infection rate pattern for Australia was specified/estimated as.

rr = c(rep(2.5,5),rep(2.3,5),rep(2.9,5),rep(3,5),rep(2.1,5),rep(1,4),rep(0.25,6),rep(0.3,10),rep(0.5,5),rep(0.2,50)) and the infection rate pattern for UK was.

rr = c(rep(3.4,10),rep(3.1,10),rep(2.2,5),rep(1.7,4),rep(1.4,6),rep(1.2,6),rep(1.1,4),rep(1,8),rep(0.9,7),rep(0.6,10),rep(0.1,30)).

Different from the model default setting for the initial cases (n0 = 1), it was decided to use the sum of the newly confirmed cases over the period of two weeks prior to 1 March as the initial infectious case number. Therefore, it was n0 = 10 for Australia data and n0 = 9 for UK data in running the simulation model. The parameter muT was also adjusted in the simulation setting to give more flexibility in fitting the observed data patterns. The simulation settings were:

TransSimu(nd = 100, muT = 4.4, sizeV = 0.9, n0 = 10) for Australia and TransSimu(nd = 100, muT = 3.8, sizeV = 1.1, n0 = 9) for UK. The resulting muT values implied that the average time needed for getting a susceptible people infected was shorter in UK than in Australia (3.8 versus 4.4). On the other hand, the resulting sizeV values implied that the infection intervals in UK was less variable than that in Australia (a higher sizeV value implied a lower variance).

**Figure 3:**
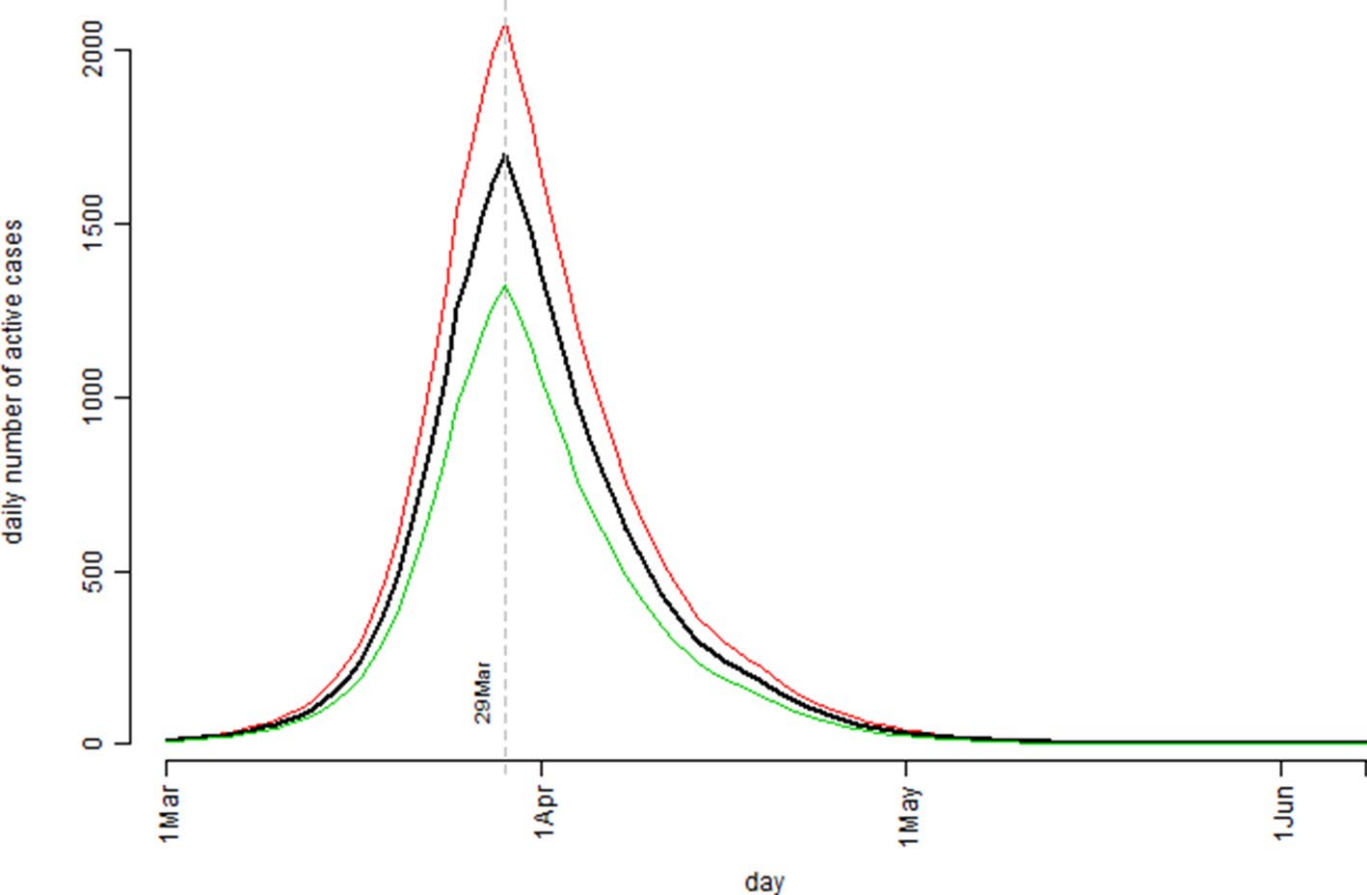
The pattern of the model estimated number of the infection active cases over the observation period based on the Australian confirmed COVID-19 cases data: the bold black curve is the median level, the thin green curve is the 25th percentile level, and the thin red curve is the 75th percentile level out of 1,000 bootstrap simulation runs.

**Figure 4:**
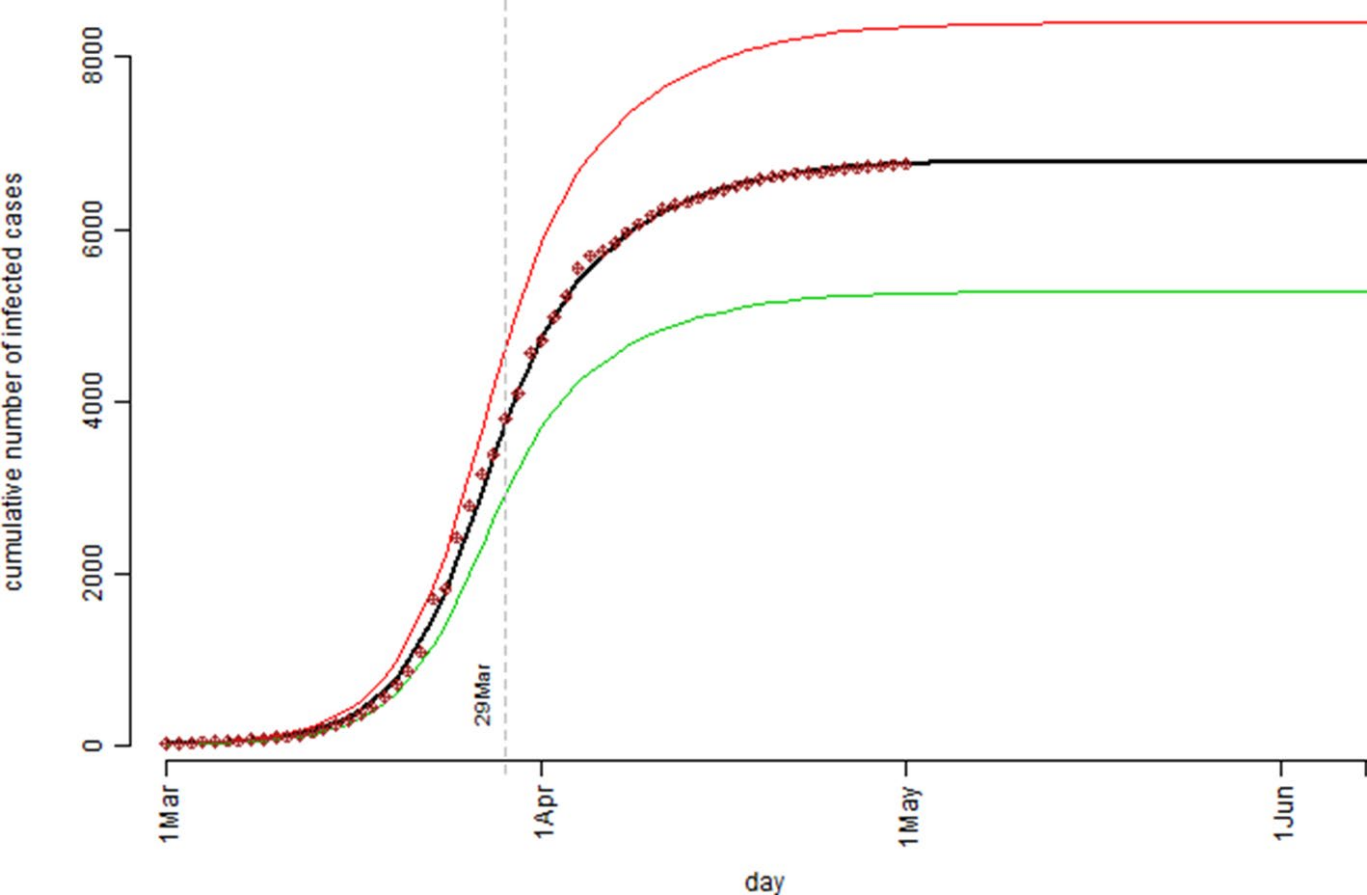
The cumulative number of the confirmed cases over the observation period for the Australian confirmed COVID-19 cases data: the bold black curve is the model estimated median level, the thin green curve is the 25th percentile level, and the thin red curve is the 75th percentile level out of 1,000 bootstrap simulation runs; the dot points are the actual recorded number of cases.

The simulation study results based on the Australian confirmed COVID-19 cases data were presented in Fig. 3 (the model estimated number of the infection active cases) and Fig. 4 (the cumulative number of the confirmed cases). Figure 3 indicated that, according to the model prediction, the number of the active cases in Australia would peak around 29 March. Practically, this would be the time the Australian health care system having the highest pressure. Decisions might then be made for preparation for the possible worst scenario situation (how much and when) as predicted by the model. Figure 4 presented the model predicted/estimated pattern for the cumulative number of cases and the actually observed/recorded number of confirmed COVID-19 cases were superimposed as those dot points. The 62 observed data points (period over 1 March to 1 May) fitted nicely to the predicted median curve. By examining the resulting Rt values, it was found that the infection rate in Australia was actually higher in later March than in early March which might be explained by the fact that a substantial large number of new cases were identified related to several cruise ships arrived in March (e.g., the Ruby Princess cruise ship case https://www.abc.net.au/news/2020-04-05/ruby-princess-cruise-coronavirus-deaths-investigated-nsw-police/12123212). As we have argued in the previous sections the determination of Rt values involved different types of influential factors and these factors were very much time dependent. Nevertheless, the simulation model predicted that at the peak time, the number of infection active cases could reach 1,670 with the interquartile range of (1,321, 2,079); by the end of the COVID-19 outbreak, it would have a total number of 6,789 confirmed cases in Australia with the interquartile range of (5,268, 8,401). Both Figs. 3 and 4 indicated that the COVID-19 outbreak in Australia should be over before June should the assumed Rt pattern in this model proved to be true. The model predicted that by 3 May the active case level in Australia would drop back to the same level prior to 1 March.

**Figure 5:**
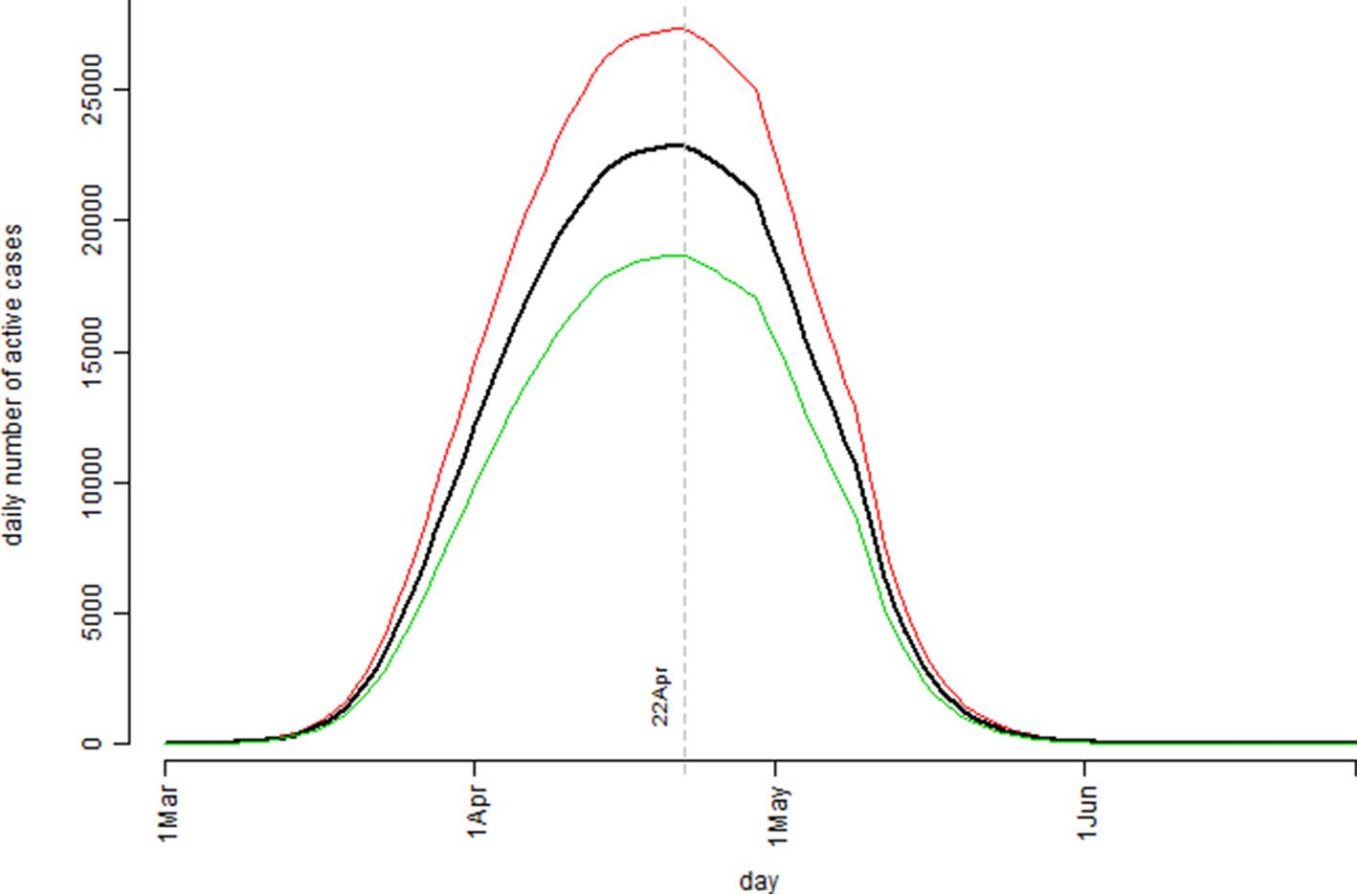
The pattern of the model estimated number of the infection active cases over the observation period based on the United Kingdom’s confirmed COVID-19 cases data: the bold black curve is the median level, the thin green curve is the 25th percentile level, and the thin red curve is the 75th percentile level out of 1,000 bootstrap simulation runs.

**Figure 6:**
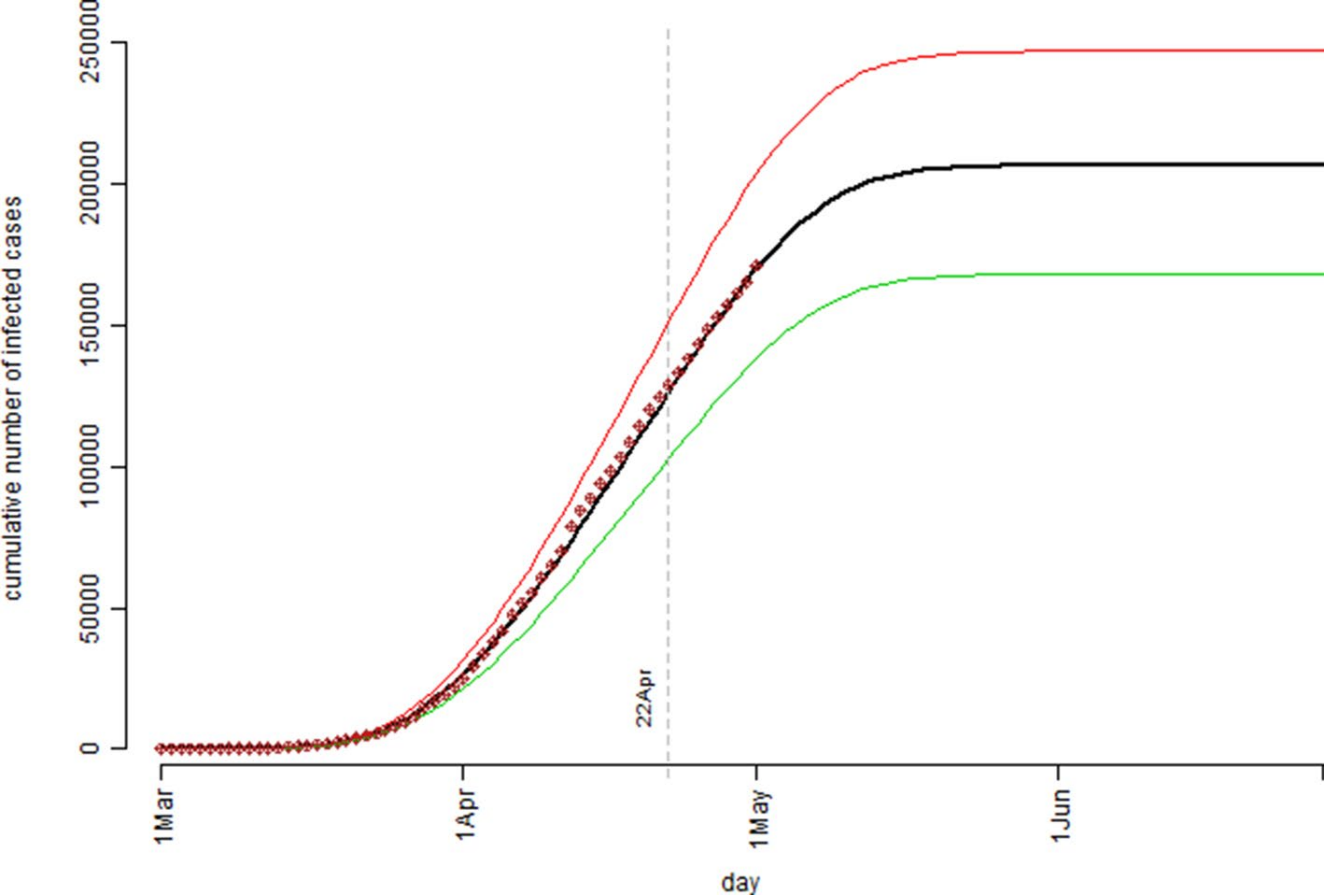
The cumulative number of the confirmed cases over the observation period for the United Kingdom’s confirmed COVID-19 cases data: the bold black curve is the model estimated median level, the thin green curve is the 25th percentile level, and the thin red curve is the 75th percentile level out of 1,000 bootstrap simulation runs; the dot points are the actual recorded number of cases.

The simulation study results based on the United Kingdom’s confirmed COVID-19 cases data were presented in Fig. 5 (the model estimated number of the active cases) and Fig. 6 (the cumulative number of the confirmed cases). Figure 5 indicated that, according to the model prediction, the number of the infection active cases in UK would peak around 22 April. The simulation model predicted that at the peak time, the number of active cases could reach 22,856 with the interquartile range of (18,653, 27,323). Practically, this would be the time the UK’s health care system having the highest pressure. For example, assuming 10% of the active case individuals would require ICU beds, this meant UK health care system should have at least 2,286 ICU beds ready before 22 April.

Same as the Australian case in Fig. 4, the UK case in Fig. 6 showed a near perfect fit between the model predicted/estimated median curve and the observed data points. The simulation model predicted that by the end of the COVID-19 outbreak, UK could have a total number of 206,481 confirmed cases with the interquartile range of (168,074, 247,047). Due to the uncertain and complex nature in determination of the Rt values, one should not be too confident about the model estimation results for both the Australia case and the UK case in terms of ultimate true cumulative number of confirmed COVID-19 cases because, as it was said “Models are only as good as the assumptions on which they are based”^15^. However, in the process of determining the Rt values for the simulation model more insights were gained on the dynamics of the COVID-19 spread over time in both countries.

The feasible interpretations of those two resulting/estimated infection rate patterns were as follows. Over 62 days (i.e., 1 March to 1 May) of the recorded data period, Australia had a much lower infection rate pattern than UK had. The details of the differences was summarised by presenting a descriptive summary of the first 62 Rt values (which primarily were determined by the observed number of confirmed cases) as in Table 2. The results should explain the reason why even both countries started with almost the same confirmed case numbers (26 versus 23 as of 1 March) but it ended with UK had as 25 times higher of the confirmed cases than Australia had (6,762 versus 171,253 as of 1 May).

**Table 2:** A numeric summary of the estimated infection rate patterns over the period 1 March to 1 May 2020.

Rt values	Minimum	25th percentile	median	mean	75th percentile	Maximum
Australia	0.2	0.25	0.5	1.25	2.3	3
UK	0.6	1.0	1.4	1.91	3.1	3.4

The infection rate patterns could also be examined by different stages over the observation period. Beginning from 1 March, the first 10 days both countries were under pre-intervention stage and the resulting/estimated infection rate was between 2.3 and 2.5 for Australia and as high as 3.4 for UK. Then the unusual changes happened for the Australian infection rates over the second 10-day in March that it increased to as high as 3. On the other hand, the UK infection rates remained steady at 3.1 over the same 10-day period. From 20 March to 5 April, the infection rates kept constantly decreasing for both countries with a sharper drop in Australia (e.g., from 3 to 0.25) than in UK (e.g., from 3.1 to 1.4). This 15-day of gradually decreasing pattern of Rt values might be interpreted as a reflection of how intervention measures were enforced and taking effect over time. Then the infection rate pattern in Australia had showed a gentle bounce-back over the period of 6 April to 20 April (e.g., from 0.25 to 0.5) while UK’s infection rates kept decreasing slowly and remained at a much higher level (e.g., from 1.4 to 1). After 20 April, both countries had showed a constantly decreasing pattern. However, caution was needed in interpretation of those Rt values after 1 May for the following reason. Of the total 100 Rt values in each pattern, the first 62 Rt values were largely determined by fitting the model to the observed data. The remaining 38 Rt values would then primarily be a result of subjective judgement or some wishful assumptions. For example, by assuming the last 30 Rt values to be 0.2 (for Australia) or 0.1 (for UK), we practically assume that, for both countries after 10 May the interventions would keep in effect, the general public would respond accordingly, and the confirmed cases keep recovering so that the overall effect was to such an extent that the infection rate would drop to a very low level of 0.2/0.1. However, what the future reality this might finally play out could only be a matter of “what will be will be”.

## Discussion

So far the hypothetical examples and the real life data models were referred to the situations in which the total number of infection cases was far below the default population size limit, one million (1,000,000). For example, Australia has a population about 25 million but the model estimated a total number of COVID-19 cases was less than 7,000; and the estimated total number of 206,450 cases versus a population size of 68 million for UK. Since COVID-19 was completely new to this world it was reasonable to assume that no immunity cohort in the current populations. In this section, however, the simulation model was examined for its performance in a different modelling situation: assuming the total infection number would reach the limit of a hypothetical population size, e.g., one million and a substantial proportion of immunity cohort, e.g., 10% (0.1).

Three scenarios characterised by three hypothetical infection rate patterns were examined. The three hypothetical infections rate patterns were specified as: rr = c(rep(5,40),rep(0.5,60)); rr = c(rep(4,40),rep(0.5,60)); rr = c(rep(3.2,40),rep(0.5,60)).

**Figure 7:**
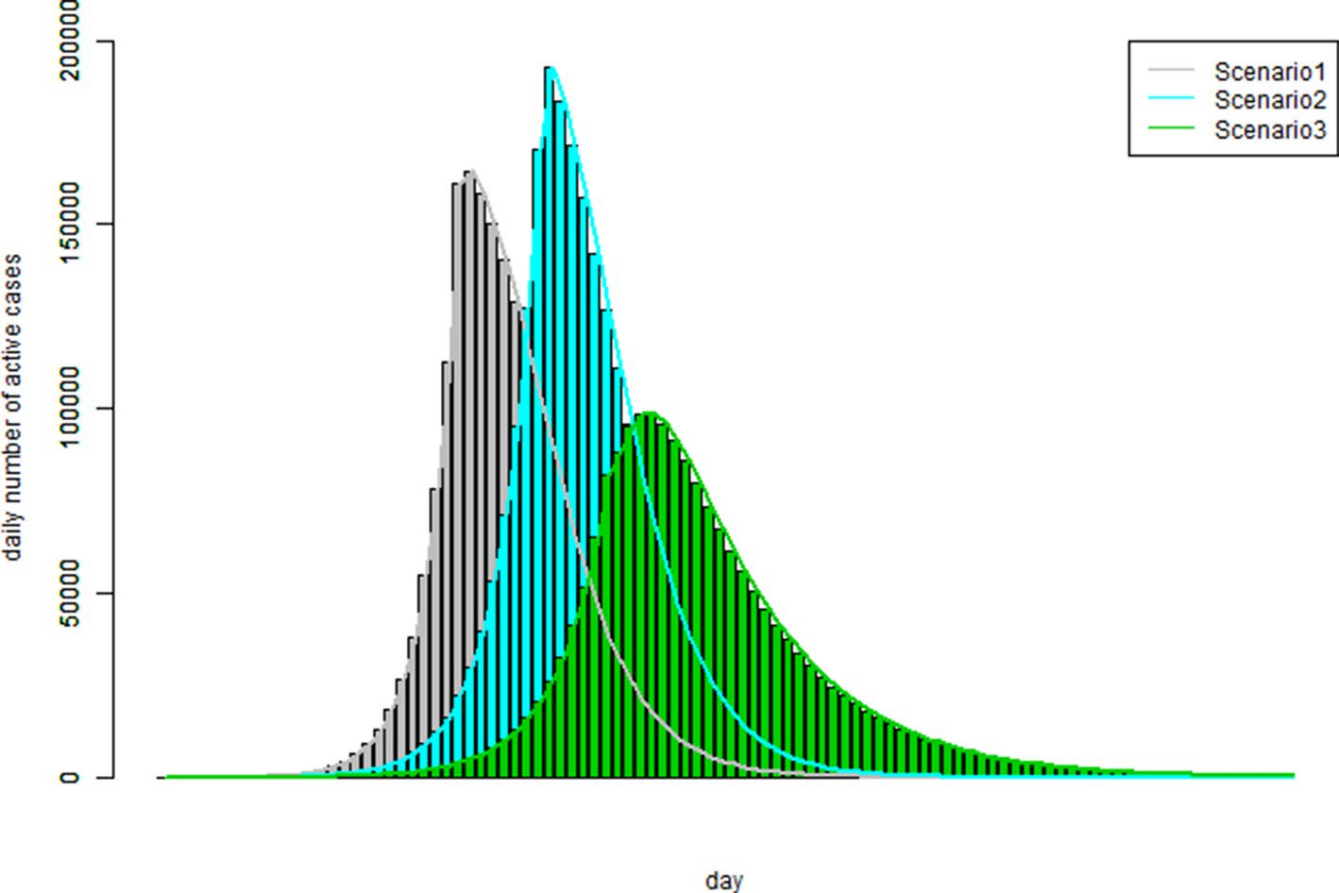
The patterns of the number of the active cases over the observation period of three hypothetical infection rate patterns which examine the impact due to population size and immunity level.

Therefore, we have assumed that in scenario 1, the infection rate was five for the first 40 days and then 0.5 for 60 days; scenario 2, infection rate was four for the first 40 days and then 0.5 for 60 days; scenario 3, infection rate was 3.2 for the first 40 days and then 0.5 for 60 days. Furthermore, we assumed that the immunity cohort proportion was 0.1 for all three scenarios. By setting the same random seed in running the simulation, we had the analysis results as graphically presented in Fig. 7.

The simulation study results showed in Fig. 7 could be interpreted as follows. In the situations that the total number of infection cases would exceed the population size (scenarios 1 and 2, which were represented by the grey and blue curves), a smaller infection rate would not necessarily widen the length of the disease outbreak period and the peak level of the number of active cases was not necessarily lower. On the other hand, if the infection rate was smaller enough so that the total number of infection cases would not reach the population size, the pattern of the number of the active cases would really flatten out as it was the case of scenario 3 (the green curve). The numeric simulation outcomes showed that this simulation model could correctly model the immunity proportion as specified in the input information.

This study had developed a novel Monte Carlo simulation procedure that could capture the essential virus transmission dynamics for the purpose of modelling COVID-19 spread over time. Through both the hypothetical and real life examples, the proposed simulation model had showed a good potential to be used as an effective and adaptable tool in performing what-if analysis for decision making for combating COVID-19 in specific and any other infectious diseases in general. With this newly developed empirical analysis tool/simulation model, however, the author is well aware of its limitations and weaknesses. For example, the current approach for estimation of the model parameters is largely an ad hoc empirical procedure. Because of its Monte Carlo simulation nature, a Bayesian approach, e.g., using one of the available BUGS language software^16,17^, would be an obvious better solution. Although, the stochastic point process model was implemented in a Monte Carlo simulation model in this study, the mathematical formulas and its theoretical properties were not established. Although a couple of ad hoc parameter sensitivity studies were performed, neither the theoretical analysis nor a systematic empirical study of the model parameter sensitivity were pursued. The author will surely deal with these identified issues in his future research.

## Supplementary information

Supplementary Information.

## Data Availability

The author confirms that the real data sets used in this study are freely available to public at the time of writing by accessing the webpage: https://ourworldindata.org/coronavirus (accessed on 7 June 2020).

## References

[1] Zhang J (2019). Evolving epidemiology and transmission dynamics of coronavirus disease 2019 outside Hubei province, China: a descriptive and modelling study. Lancet Infect. Dis..

[2] Roser, M., Ritchie, H., Ortiz-Ospina, E. & Hasell, J. Coronavirus pandemic (COVID-19) (2020)

[3] Ciarochi, J. *Modeling Infectious Diseases*, vol. 2020. https://triplebyte.com/blog/modeling-infectious-diseases (2020).

[4] Fitzpatrick MC, Bauch CT, Townsend JP, Galvani AP (2019). Modelling microbial infection to address global health challenges. Nat. Microbiol..

[5] Sattenspiel L (1990). Yearbook of Physical Anthropology.

[6] Xie, G. *Further developments of two point process models for fine-scale time series*. Doctor of Philosophy thesis, Massey University, Wellington (2011).

[7] Cox D, Isham V (1980). Point Processes.

[8] Team RC (2019). R: A Language and Environment for Statistical Computing (Version 3.6.2).

[9] Ferguson N (2020). Impact of Non-pharmaceutical Interventions (NPIs) to Reduce COVID19 Mortality and Healthcare Demand.

[10] Casella G, Berger RL (2002). Statistical Inference.

[11] Krishnamoorthy K (2006). Handbook of Statistical Distributions with Applications.

[12] Crawley MJ (2013). The R book.

[13] Efron B, Tibshirani RJ (1993). An Introduction to the Bootstrap.

[14] He X (2020). Temporal dynamics in viral shedding and transmissibility of COVID-19. Nat. Med..

[15] Wikipedia. (2020).

[16] Gelman A (2014). Bayesian Data Analysis.

[17] Lunn D, Jackson C, Best N, Thomas A, Spiegelhalter D (2012). The BUGS Book: A Practical Introduction to Bayesian Analysis.

