# Supplementary material for: A novel Monte Carlo simulation procedure for modelling COVID-19 spread over time
Source: Sci Rep. 2020 Aug 4;10:13120. doi: 10.1038/s41598-020-70091-1 (PMC7403316; doi:10.1038/s41598-020-70091-1)
Supplement: Supplementary file 1 — Supplementary Information. [file 41598_2020_70091_MOESM1_ESM.docx]

A novel Monte Carlo simulation procedure for modelling COVID-19 spread over time

Author: Gang Xie *

Email: [gxie@csu.edu.au](mailto:gxie@csu.edu.au)

Affiliation: Research Office, Charles Sturt University, Wagga Wagga, NSW, Australia

Declarations:

- The author declares no competing interests.
- The author confirms that the real data sets used in this study are freely available to public at the time of writing by accessing the webpage: <https://ourworldindata.org/coronavirus> (accessed on 7 June 2020)
- The author received no external funding for this research.

**Appendix**: R code of the Monte Carlo simulation model for representing the dynamics of the COVID-19 spread processes.

#---------------------------------------------------------------------------------------------------------------------

TransSimu = function(days = 300, nd = 30, Rt = rr, muT = 4, sizeV = 1,limit=1000000, pp=0.001,n0=1)

{ # beginning of the function

# days: observation period

# nd: simulation period

# Rt = rr # infection rate pattern

# muT is the mean time an infected person will transmit the virus to (i.e., infect) another person.

# We assume that the independence among those ones being infected. The default value is set as muT = 4 (days).

# sizeV: the dispersion parameter so that variance = mu + mu^2/size. The default value is set as sizeV =1.

# limit: the target/study population size

# pp: the proportion of people with immunity in the population

# n0: the initial number of infectious persons.

# The default setting assumes one virus carrier/infectious person in the beginning, i.e., n0=1.

#

kk = atrisk = rep(0,days); nn = length(kk)

# kk: daily new cases; atrisk: number of active cases each day; simulation period of nn days

tt = 0 # the cumulative total number of confirmed cases.

if(nd > length(Rt)) stop("The length of Rt should not be smaller than nd.")

stoplimit = limit*(1-pp)

nk = n0 # The initial number of existing infectious persons.

# there must be a first patient to kick off the transmission process!

for(k in 1:nk) {

#

if(tt>stoplimit) Rt[1]=0.001

ni = rpois(1,Rt[1]) # how many people will be infected by this existing virus carrier person.

imuind = sample(c(0,1), 1, prob=c((1-pp),pp))

if(imuind==1) ni=0

tt=tt+ni

if(ni > 0) {

tk = rep(0,ni)

for (i in 1:ni) {

tk[i] = rnbinom(1,size=sizeV,mu=muT)+1 # this is the nth day on which a new case occurs

kk[tk[i]] = kk[tk[i]] + 1

}

#

pastevent = c(rep(1,(max(tk)-1)),rep(0, (days-max(tk)+1)))

atrisk = atrisk + pastevent

} # end of if(ni > 0)

} # end of k loop

#

for(j in 2:nd) {

nk = kk[j-1] # this is the number of people newly infected (i.e., new cases) on (j-1)th day

if(nk > 0) {

for(k in 1:nk) {

#

if(tt>stoplimit) Rt[j]=0.001

ni = rpois(1,Rt[j]) # how many people will be infected by this existing virus carrier person.

imuind = sample(c(0,1), 1, prob=c((1-pp),pp))

if(imuind==1) ni=0

tt=tt+ni

if(ni > 0) {

tk = rep(0,ni)

for (i in 1:ni) {

tk[i] = rnbinom(1,size=sizeV,mu=muT)+1+j # this is the nth day on which a new case occurs

kk[tk[i]] = kk[tk[i]] + 1

}

#

pastevent = c(rep(0, (j-1) ), rep(1,(max(tk)+1-j)),rep(0, (days-max(tk))))

atrisk = atrisk + pastevent

} # end of if(ni > 0)

} # end of k loop

} # end of if(nk > 0)

#

} # end of j loop

list(riskpopu = atrisk, dailynew = kk, total=tt) # output information

} # end of the function

#------------------------------------------------------------------------

# observed number of confirmed infection cases in Australia and UK over the period of 1 March to 1 May 2020

# data source: https://ourworldindata.org/coronavirus

# oz = Australia; uk = United Kingdom

oz = c(26, 29, 33, 41, 52, 59, 63, 74, 80, 100, 112, 126, 156, 197, 249,

298, 375, 454, 565, 709, 874, 1098, 1709, 1823, 2423, 2799, 3166, 3378, 3809,

4093, 4557, 4707, 4976, 5224, 5548, 5687, 5744, 5844, 5956, 6052, 6152, 6238, 6289,

6322, 6366, 6416, 6458, 6497, 6533, 6586, 6612, 6625, 6647, 6654, 6667, 6687, 6703,

6713, 6725, 6738, 6746, 6762)

uk = c(23, 36, 40, 51, 85, 115, 163, 206, 273, 321, 373, 456, 590, 707, 1140, 1391, 1543,

1950, 2630, 3277, 3983, 5018, 5683, 6650, 8077, 9529, 11658, 14543, 17089, 19522,

22141, 25150, 29474, 33718, 38168, 41903, 47806, 51608, 55242, 60733,

65077, 70272, 78991, 84279, 88621, 93873, 98476, 103093, 108692, 114217,

120067, 124743, 129044, 133495, 138078, 143464, 148377, 152840, 157149, 161145,

165221, 171253)

#---------------------------------

rr = c(rep(2.5, 5), rep(2.3, 5), rep(2.9, 5), rep(3, 5), rep(2.1, 5), rep(1, 4), rep(0.25, 6), rep(0.3, 10), rep(0.5, 5), rep(0.2, 50)) # resulting/estimated infection rate over time pattern for Australia

rr = c(rep(3.4,10), rep(3.1,10), rep(2.2, 5),rep(1.7, 4),rep(1.4, 6), rep(1.2, 6), rep(1.1, 4), rep(1, 8), rep(0.9, 7),rep(0.6,10),rep(0.1, 30))

# resulting/estimated infection rate over time pattern for UK

#---------------------------------

# A bootstrap procedure for the point estimates and interval estimates:

set.seed(10)

outMA = newMA = NULL; gtotalA = NULL

for(m in 1:1000) {

# runi = TransSimu(nd=100, muT=4.4,sizeV=0.9,n0=10) #oz 1 May

runi = TransSimu(nd=100, muT=3.8,sizeV=1.1,n0=9) #uk 1 May

this = runi$riskpopu[1:150]

outMA = rbind(outMA, this)

thisnew = runi$dailynew[1:150]

newMA = rbind(newMA, thisnew)

gtotalA = c(gtotalA, runi$total)

}

#---------------------------------------------------------------------------
